# Supplementary material for: Validity and Reliability of the Staden Schizophrenia Anxiety Rating Scale
Source: Diagnostics (Basel). 2022 Mar 25;12(4):831. doi: 10.3390/diagnostics12040831 (PMC9028449; doi:10.3390/diagnostics12040831)
Supplement: Supplementary file 1 [file diagnostics-12-00831-s001.zip › diagnostics-1648432-supplementary.pdf]

Patient | | | | Date: | |

Staden Schizophrenia Anxiety Rating Scale (S-SARS)

WERDIE VAN STADEN MBCHB, MMED(PSYCH), MD, FCPSYCH(SA), FTCL, UPLM

SPECIFIC ANXIETY

1. Persecutory and nihilistic anxiety

0	Absent	No persecutory or nihilistic anxiety during the past 7 days.
1	Questionably present	Unclear whether persecutory or nihilistic anxiety has been present or absent.
2	Mild	The patient has been concerned about the attitude, intentions, or plans of other beings towards him/her, **or** the patient is afraid that life has lost its meaning for him/her.
3	Moderate	The patient is afraid that he/she *may* be persecuted, **or** *may* be a victim of malicious intent, **or** the patient is afraid that his/her life or livelihood is drawing to an end.
4	Moderately severe	The patient is afraid as part of his/her belief that something bad or harmful *is about to be done* to him/her, **or** the patient is afraid as part of his/her belief that his/her life is in danger.
5	Severe	The patient is afraid as part of his/her belief that something bad or harmful *is being done* or *was done* to him/her, **or** the patient is afraid as part of his/her belief that he/she is dying, decaying, or ceasing to exist fully.

Guidelines:

Consider all information pertaining to delusions, if present, for potential relevance.

Consider all information pertaining to self-worth and appraisal of own resources (livelihood) for potential relevance.

Minimum enquiries:

During the past week, have you been concerned about the attitude of someone towards you?

Have you been concerned about someone’s intentions or plans that involve you?

Who do you trust least in your life?

Are you concerned that someone may be against you?

Are you afraid that life has lost its meaning for you?

Are you afraid that someone may be out to get you?

Are you afraid that someone may be about to harm you?

Is your livelihood (sources for living) drawing to an end?

Is your life in danger?

Are you fearing for your life or future existence?

Patient | | | | Date: | |

2. Perceptual anxiety

0	Absent	No anxiety related to sensory perceptions during the past 7 days.
1	Questionably present	Unclear whether anxiety related to sensory perceptions has been present or absent.
2	Mild	The patient has been *concerned* about things or people he/she hears, sees, feels tactually, smells or tastes, **and** these sensory perceptions are dreams, images, illusions, or hallucinations.
3	Moderate	The patient has been *afraid* of the things or people he/she hears, sees, feels tactually, smells or tastes, **and** these sensory perceptions are dreams, images, illusions, or hallucinations.
4	Moderately severe	The patient has been *afraid* **and** has been *startled* by the things or people he/she hears, sees, feels tactually, smells or tastes, **and** these sensory perceptions are dreams, images, illusions, or hallucinations.
5	Severe	The patient has been afraid or scared when objectively hallucinating.

Guidelines:

Consider all information pertaining to dreams, images, illusions & hallucinations, if present.

Minimum enquiries:

During the past week, have you been concerned about your dreams? If so, have you been afraid of what you saw or heard or smelled or felt on you skin in your dreams?

Have you seen any images that caused concern? Or fear?

Have you been startled lately?

Have you seen or heard anything distorted?

Have you seen or heard anything and someone else disagreed about what it actually had been?

Has someone communicated verbally with you, without being visibly present (excluding a phone or similar)?

Have you heard something that other people could not hear?

Have you seen something that other people could not see?

Have you smelled something that other people could not smell?

Have you tasted something without anything being in your mouth, or touching your tongue?

Have you felt something on your skin that other people could not see?

Have you been concerned about *what* you have heard? Or have seen, smelled, tasted, felt on your skin?

Have you been afraid about *what* you heard? Or have seen, smelled, tasted, felt on your skin?

3. Anxiety attacks

0	Absent	No anxiety attacks during the past 7 days.
1	Questionably present	Unclear whether anxiety attacks have been present or absent.
2	Mild	The patient has had at least one discreet episode of intense fear **without** much concern about a further attack or its cause or its implications.
3	Moderate	The patient has had at least one discreet episode of intense fear, **and** has been concerned about a further episode or its cause or its implications.
4	Moderately severe	The patient has had *more than one* discreet episode of intense fear **and** has been very concerned about a further episode or its cause or its implications.
5	Severe	The patient has had *more than one* discreet episode of intense fear, **and** has been very concerned that these episodes may be an indication of his/her impending death.

Guidelines:

Consider information pertaining to any discreet episodes of intense fear, even if not a "panic attack" in the strict sense, and even if the cause of the fear is known.

Minimum enquiries:

Have you experienced intense fear during the past week?

[If so] How many times?

Have you been concerned that it would happen again?

Have you been concerned about the cause of this/these episode(s) of fear?

Have you been concerned about the implications of this/these episode(s)?

Have you been concerned that these episodes may be an indication of impending death?

Patient | | | | Date: | |

4. Situational anxiety

0	Absent	No situational anxiety during the past 7 days.
1	Questionably present	Unclear whether situational anxiety has been present or absent.
2	Mild	The patient has experienced discomfort in any one such situation **or** has been avoiding any one such situation.
3	Moderate	The patient has experienced discomfort in any one such situation, **and** has been avoiding any one such situation.
4	Moderately severe	The patient has experienced marked distress in any one such situation **or** has taken excessive measures to avoid any one such situation.
5	Severe	The patient has experienced marked distress in one such situation, **and** has taken excessive measures to avoid any one such situation.

Guidelines:

Enquire about the following or similar situations during the past 7 days:

Being with familiar people;
being with strangers;
talking to strangers;
being in a group;
talking in front of a group;
eating in a group;
being alone;
being in crowded shops, busy streets, closed-in spaces (tunnels, bridges, elevators);
and closed-in vehicles (cars, buses, taxis, trains, aeroplanes).

Rate only if the patient had anxiety in such a situation, or if the patient has been avoiding such a situation

Minimum enquiries:

During the past week, have you experienced discomfort in …[ask **in turn** about each of the above situations]?

[If so] Have you experienced marked distress in that situation?

Have you been avoiding … [ask **in turn** about each of the above situations]?

[If so] Have you gone out of your way to avoid that situation?

Patient | | | | Date: | |

5. Obsessive-compulsive anxiety

0	Absent	No obsessive-compulsive anxiety during the past 7 days.
1	Questionably present	Unclear whether obsessive-compulsive anxiety has been present or absent.
2	Mild	*At times*, the patient has had repetitive and intrusive thoughts and/or behaviour.
3	Moderate	*Often*, the patient has had repetitive and intrusive thoughts and/or behaviour.
4	Moderately severe	*For at least half of the time*, the patient has been occupied with repetitive and intrusive thoughts and/or behaviour.
5	Severe	*For at least half of the time*, the patient has been occupied with repetitive and intrusive thoughts and/or behaviour, **and** for most of the time he/she does not attempt to resist these thoughts and/or behaviour.

Guidelines:

Enquire about the following (or similar) repetitive and intrusive thoughts and behaviours during the past 7 days:

Thoughts about contamination;
the need for symmetry;
the need for a specific order;
the need to have things "just right";
or somatic concerns;
sexual or perverse thoughts;
aggressive thoughts;
excessive doubt;
checking behaviour;
cleaning; counting;
need to ask or confess;
rituals;
ordering or arranging items symmetrically or precisely;
hoarding items.

Rate only thoughts associated with anxiety and which are recognised by the patient as his/her own (e.g. not the thoughts of "thought insertion").

Do not rate thoughts that are merely worries about real-life problems.

Minimum enquiries:

During the past week, have you had…[ask **in turn** about each of the above thought and behaviours]?

[If so] Has this thought or behaviour been intrusive?

[If so] Has it intruded a few times, often, or at least half of the time?

[If at least half of the time] Have you attempted to resist it?

Patient [| |] Date: [ ]

General Anxiety

6. Somatic anxiety

0	Absent	No somatic anxiety during the past 7 days.
1	Questionably present	Unclear whether somatic anxiety has been present or absent.
2	Mild	One or more of these experiences have been present, but were neither particularly distressful nor disruptive for the patient.
3	Moderate	One or more of these experiences were disruptive or distressful for the patient.
4	Moderately severe	More than one of these experiences have been so distressful or disruptive that the patient has been fearing a re-occurrence **or** the patient has avoided situations or other potential precipitants of these experiences.
5	Severe	More than one of these experiences have been unbearable for the patient **and** disrupted many of his/her daily activities.

Guidelines:

Enquire about ***each*** *of the following experiences*:

Palpitations;
tachycardia;
shortness of breath;
feeling pressure on chest;
feeling tense;
headaches;
tense muscles;
sweating excessively;
trembling;
giddiness or dizziness;
(non-Parkinsonian) tremors

Rate these irrespective of cause

Minimum enquiries:

During the past week, have you had…[ask **in turn** about each of the above experiences]?

[If so] Has it (or have they) been disruptive or distressful to you?

[If so] Have you been fearing a re-occurrence of them?

Have you avoided situations or other potential triggers of them.

Have they been unbearable to you?

Have they disrupted many of your daily activities?

Patient | | | | Date: | |

7. Psychomotor and cognitive agitation

0	Absent	No psychomotor or cognitive agitation during the past 7 days.
1	Questionably present	Unclear whether psychomotor or cognitive agitation has been present or absent.
2	Mild	A few times, the patient has been restless, or could not sit still, or has been pacing
3	Moderate	Several times, the patient has been restless, or could not sit still, or has been pacing.
4	Moderately severe	Several times, the patient has been restless, or could not sit still, or has been pacing **and** was *observed* to be startled easily.
5	Severe	The patient has been restless, or could not sit still, or has been pacing, **and** the patient has been *observed* to be overly distractible or has been *observed* to have poor concentration.

Guidelines:

Consider information provided by the patient, professional staff and other care-givers. The interviewer should also include his/her own observations during the interview and the past week.

Minimum enquiries:

During the past week, have you been restless a few times?

Could you not sit still at times?

Have you been pacing a few times?

Has it happened several times?

Please say the months of the year backwards?

Please subtract 7 from 100 and continue subtracting 7 from each answer (The interviewer should not assist or confirm after the second answer)

Patient [| |] Date: [ ]

8. Worry and fear

0	Absent	No worries or fears during the past 7 days.
1	Questionably present	Unclear whether worries or fears have been present or absent.
2	Mild	The patient has been worried *at times* about a (trivial or important) problem(s), or about himself or herself, or about someone else, or about his/her/others' affairs.
3	Moderate	The patient has been worried *often*, **or** has been afraid that something *unwanted* would happen or has already happened.
4	Moderately severe	The patient has been worried *continuously*, **or** has been afraid that something *bad* is about to happen to him/her or someone else.
5	Severe	The patient has been afraid that he/she or someone else would die or be seriously harmed in the near future.

Guidelines:

Consider all information pertaining to worry and fear as revealed through out the interview, and information provided by professional staff and other care-givers.

Include all worries and fears whether about real-life problems, or about supposed problems or threats.

Minimum enquiries:

During the past week, have you been worried about yourself at times?
Have you been worried about your affairs at times?
Have you been worried about someone else, or about his/her affairs at times?
Have you been worried about anything else?
Have you been worried often, or perhaps continuously?
Have you afraid that something unwanted would happen or has already happened?
Have you afraid that something bad would happen or has already happened?
Have you been afraid that you or someone else would die or be seriously harmed in the near future?

9. Control-related anxiety

0	Absent	No concerns about control in the patient's life during the past 7 days.
1	Questionably present	Unclear whether concerns about control in the patient's life have been present or absent.
2	Mild	The patient has been concerned about not having control, **or** has been concerned about having too much to control in his/her life.
3	Moderate	The patient feared losing control in his/her life, **or** the patient has felt out of control of his/her life.
4	Moderately severe	The patient has feared that he/she would be controlled or taken over.
5	Severe	The patient has been scared by the experience that he/she or his/her thoughts or life is controlled or taken over by someone or something.

Guidelines:

Rate irrespective of whether concerns about control are of delusional nature or based on perceptual disturbances

Minimum enquiries:

During the past week, have you been concerned about a lack of control in your life?
Have you been concerned about having too much to control in your life?
Have you feared losing control?
Have you felt out of control of your life?
Have you feared that you would be controlled or taken over?
Have you experienced that someone or something controls or has taken over your thoughts or your life? If so, has that scared you?

Patient | | | | Date: | |

10. Impairment from anxiety

0	Absent	No impairment from anxiety during the past 7 days.
1	Questionably present	Unclear whether impairment from anxiety has been present or absent.
2	Mild	*At times*, anxiety has prevented the patient from doing things he/she could ordinarily do, **or** anxiety has prevented him/her from sleeping properly.
3	Moderate	*Often*, anxiety has prevented the patient from doing things he/she could ordinarily do, **or** anxiety has prevented him/her from sleeping properly.
4	Moderately severe	*For at least half of the time*, the patient has been avoiding people or situations owing to fears about them.
5	Severe	*For at least half of the time*, anxiety has prevented the patient from doing things he/she could ordinarily do, **and** the patient has been avoiding people or situations owing to fears about them.

Guidelines:

Consider also impairment related to ALL the other items (9) of this rating scale.

Include both clinical judgments and the subjective judgments by the patient

Minimum enquiries:

During the past week, for at least half of the time, have you been avoiding people or situations owing to fears about them?
Have concerns, worries or fears kept you from sleeping properly?
Have you been too restless or agitated to sleep?
Have concerns, worries, fears, or restlessness prevented you from doing things you could ordinarily do? If so, has it been at times, often, or for at least half of the time?

TOTAL SCORE: [ ]
